# ﻿*Lijiangomyceslaojunensis* gen. et sp. nov. (Mytilinidiaceae), and *Sclerococcumstictae* (Dactylosporaceae), a new lichenicolous species from Yunnan, China

**DOI:** 10.3897/mycokeys.114.146031

**Published:** 2025-03-04

**Authors:** Qingfeng Meng, Paul Diederich, Vinodhini Thiyagaraja, Damien Ertz, Xinyu Wang, Natsaran Saichana, Kevin D. Hyde, Ruvishika S. Jayawardena, Shaobin Fu

**Affiliations:** 1 School of Public Health, Zunyi Medical University, Zunyi City, Guizhou Province 563000, China; 2 Center of Excellence in Fungal Research, Mae Fah Luang University, Chiang Rai 57100, Thailand; 3 School of Science, Mae Fah Luang University, Chiang Rai 57100, Thailand; 4 Musée national d'histoire naturelle, 25 rue Munster, L - 2160 Luxembourg, Luxembourg; 5 Key Laboratory of Phytochemistry and Natural Medicines, Kunming Institute of Botany, Chinese Academy of Sciences, Kunming 650201, China; 6 Meise Botanic Garden, Research Department, Nieuwelaan 38, 1860 Meise, Belgium; 7 Fédération Wallonie-Bruxelles, Service Général de l'Enseignement Supérieur et de la Recherche Scientifique, rue A. Lavallée 1, 1080 Bruxelles, Belgium; 8 Yunnan Key Laboratory for Fungal Diversity and Green Development, Kunming Institute of Botany, Chinese Academy of Sciences, Kunming 650201, China; 9 Kyung Hee University, 26 Kyungheedae-ro, Dongdaemun-gu, Seoul 02447, Republic of Korea; 10 School of Pharmacy, Zunyi Medical University, Zunyi City, Guizhou Province 563000, China

**Keywords:** 2 new taxa, lichen, morphology, phylogeny, saprobe, taxonomy

## Abstract

*Lijiangomyceslaojunensis***gen. et sp. nov.** and *Sclerococcumstictae***sp. nov.** are reported from China and identified through DNA sequence analyses (LSU, ITS, and *tef*1-α) and morphological characteristics. Phylogenetic analysis showed that *L.laojunensis* forms a distinct lineage within Mytilinidiaceae, closely related to the *Mytilinidion* subclade, leading to the establishment of a new genus within this family. This saprotrophic species grows on the bark of *Abiesfabri*, often surrounded by the thallus of *Pertusaria* species. *Lijiangomyceslaojunensis* is characterized by broadly open, black hysterothecia, clavate asci, and uniseriate, hyaline muriform ascospores. *Sclerococcumstictae*, a new lichenicolous species, forms a sister clade relationship to a lichenicolous fungus, *S.ricasoliae*. It was found on the thallus of *Sticta*, and is characterized by black apothecia, elongate, cylindrical asci, and brown, elliptical, and 1-septate ascospores. Descriptions, illustrations, and phylogenetic analysis results of the new taxa are provided.

## ﻿Introduction

Mytilinidiaceae belongs to Mytilinidiales, Dothideomycetes (Hyde et al. 2024), and was introduced by Kirschstein (1924) to accommodate four genera that were originally classified under Hysteriaceae. Boehm et al. (2009a) introduced Mytilinidiales based on multigene phylogenetic analysis to accommodate Mytilinidiaceae. Pem et al. (2024) included eight genera within Mytilinidiaceae: *Actidium*, *Lophium*, *Mytilinidion*, *Ostreola*, *Peyronelia*, *Pseudocamaropycnis*, *Quasiconcha* and *Zoggium*. Hyde et al. (2024) added three more genera: *Bullatosporium*, *Camaroglobulus* and *Halokirschsteiniothelia*, increasing the total to eleven genera within this family. Members of Mytilinidiaceae are distinguished by their globose or obovoid pseudothecia which are typically oyster- or hatchet-shaped with a longitudinal keel and crested apex. They feature bitunicate asci containing eight ascospores that are arranged uniseriately, biseriately, or in aggregated clusters. The ascospores are hyaline to brown, exhibiting diverse morphologies such as scolecospores, didymospores, phragmospores, or dictyospores, and with bipolar symmetry (Boehm et al. 2009a).

*Mytilinidion* was established to accommodate the type species *M.aggregatum*. This genus is characterized by globoid to obovoid, erect, conchate, or dolabrate ascomata, and a thin-walled and peridium, bitunicate, 8-spored asci. The ascospores are hyaline to brown and transversely 3–5(–7)-septate (Boehm et al. 2009b; Jayasiri et al. 2018). *Ostreola* was introduced to accommodate two species, *O.consociate* and *O.sessilis*, characterized by conchiform to hatchet-shaped hysterothecia with a longitudinal slit that opens narrowly. The asci are cylindrical, containing eight uniseriate, brown and muriform ascospores (Darker 1963). *Ostreola* shares similarities with *Mytilinidion* and *Lophium* in the ascomatal morphology but is distinguished by the presence of muriform ascospores. Lumbsch and Huhndorf (2007) placed *Ostreola* within Mytilinidiaceae. The members of *Mytilinidion* are primarily saprobic and plant pathogens, while *Ostreola* species are primarily saprobic.

*Sclerococcum* belongs to Dactylosporaceae, Sclerococcales, Eurotiomycetes (Hyde et al. 2024), and was described by Fries (1819, 1825) to accommodate the parasitic hyphomycetous fungus *S.sphaerale*, which was previously classified as *Spilomasphaerale*. Phylogenetic analysis by Diederich et al. (2013) revealed that *S.sphaerale* clusters with two species of *Dactylospora*. In a subsequent study incorporating expanded molecular data, Diederich et al. (2018) proposed adopting the family name Dactylosporaceae, with Sclerococcaceae as its synonym. They further recommended retaining *Sclerococcum* as the valid genus name and synonymizing *Dactylospora* under it, as *Sclerococcum* has nomenclatural priority over *Dactylospora*. Consequently, 46 *Dactylospora* species were transferred to *Sclerococcum* (Diederich et al. 2018). Dactylosporaceae comprises six genera: *Cylindroconidiis*, *Fusichalara*, *Gamsomyces*, *Pseudosclerococcum*, *Rhopalophora* and *Sclerococcum* (Hyde et al. 2024).

During a survey of microfungi in Yunnan Province (Thiyagaraja et al. 2024), two unidentified fungal specimens were collected. The first specimen was found on the bark of *Abiesfabri*, and was often surrounded by the thallus of *Pertusaria* species while the second was observed growing on the thallus of a foliose lichen belonging to *Sticta*. Phylogenetic analyses and morphological comparison reveal that the first specimen represents a novel genus closely related to *Mytilinidion* within Mytilinidiaceae. The second specimen is proposed as a new lichenicolous species within the genus *Sclerococcum* based on morpho-molecular analyses.

## ﻿Materials and methods

### ﻿Sample collection and morphological examination

The specimens were collected from Yunnan Province, China and the important collection information was noted (Rathnayaka et al. 2024). Macro-morphological characteristics were observed using a stereomicroscope (Olympus ZX-16) and photographed with a fitted digital camera (Olympus SC180). The ascomata were sliced, temporarily mounted, and observed under a compound microscope (Nikon Y-TV55), with images captured using a fitted digital camera (Nikon DS-Ri2). Distilled water was used as a mounting slide solution and 10% potassium hydroxide (K) and Lugol’s iodine solution (I) were used to stain and examine the hymenium. Photographic plates were assembled using Adobe Photoshop CC 2019 software (Adobe Systems, USA). Measurements were conducted using Image Framework software (Tarosoft, Version 0.9.7). The length, width, and length/width ratio (l/w) of asci and ascospores are provided (where n ≥ 10) as: (min–) [X−SD]–[X+SD] (–max), where “min” and “max” represent the extreme observed values, X is the arithmetic mean, and SD is the standard deviation. The number of measurements (n) is indicated. Unless otherwise specified, measurements were taken from water mounts, and procedures followed those specified by Senanayake et al. (2020). The holotype specimens are deposited in the Lichen Herbarium of Kunming Institute of Botany (KUN-L), Chinese Academy of Science, Yunnan, China.

### ﻿DNA extraction, PCR amplification, and sequencing

Apothecia were carefully removed with a razor blade under a dissecting microscope, and the lichen thallus was thoroughly cleaned. The sample was then transferred to a 200 μL centrifuge tube. Total genomic DNA was extracted using a Forensic DNA Kit (Omega Bio-Tek, Norcross, Georgia), following the manufacturer’s instructions. The primer pairs ITS1f/ITS4, LR0R/LR5, 983F/2218R and mrSSU1/mrSSU3R were used respectively, to amplify the internal transcribed spacer region of rDNA (ITS), the 28S large-subunit of rDNA (LSU), the translation elongation factor 1-alpha (*tef*1-α), and the mitochondrial small subunit ribosomal RNA gene (mtSSU) (Vilgalys and Hester 1990; White et al. 1990; Gardes and Bruns 1993; Zoller et al. 1999; Rehner and Buckley 2005).

The polymerase chain reaction (PCR) was performed using a Mastercycler (Bio-RAD T-100) in a 25-μL reaction volume consisting of 12.5 μL of 2 × Mix (Solarbio, dNTPs Mix), 9.5 µL of double-distilled water (ddH_2_O), 1.0 µL of each primer (10 mM), and 1.0 µL of the DNA template. The PCR conditions were as follows: an initial denaturation at 95 °C for 3 minutes, followed by 35 cycles of denaturation at 95 °C for 45 seconds, annealing at 53 °C for ITS, LSU, and mtSSU or at 58 °C for *tef*1-α, for 90 seconds, and elongation at 72 °C for 1 minute. A final extension was performed at 72 °C for 10 minutes, and the reaction was then held at 4 °C indefinitely. PCR products were sequenced by Shanghai Sangon Biotech (Chengdu, Sichuan Province, China).

### ﻿Sequence alignment and phylogenetic analyses

The quality of chromatogram sequences was verified using BioEdit Sequence Alignment software (Version 7.0.9.0). Forward and reverse sequences were assembled with ContigExpress software (New York, USA). The newly generated sequences were subjected to BLASTn searches (https://blast.ncbi.nlm.nih.gov/Blast.cgi) and deposited in the GenBank database. BLAST analysis of three genes (LSU, ITS, and *tef*1-α) from specimen KUN-L 88703 indicated that this species might belong to Gloniales, Hysteriales, or Mytilinidiales. To confirm its phylogenetic placement, all available sequence data from these orders were downloaded from GenBank (Table 1). Additionally, representative species from six adjacent orders, *viz.*Botryosphaeriales, Capnodiales, Dothideales, Jahnulales, Patellariales, and Pleosporales were included based on references from Boehm et al. (2009b). Two species of *Orbilia* (Orbiliomycetes, Orbiliales, Orbiliaceae) were selected as the outgroup taxa following Hongsanan et al. (2020) for phylogenetic analysis.

**Table 1.** Sequences used in phylogenetic analysis of Mytilinidiales and adjacent orders, with specimens or strains information and GenBank accession numbers. Newly obtained sequences are in bold font. “NA” indicates the sequence is unavailable.

Species name	Voucher/strains	GenBank accession numbers		
		ITS	LSU	*tef*1-α
*Aigialusgrandis*	BCC 20000	NA	GU479775	NA
*A.mangrovis*	BCC 33563	NA	GU479776	GU479840
*A.parvus*	PUFD45	MK028710	MK026761	MN520611
*A.rhizophorae*	BCC 33572	NA	GU479780	GU479844
*Aliquandostipitecrystallinus*	AF007	NA	EF175652	NA
*A.khaoyaiensis*	MFLUCC 21-0106	MT864350	MT860428	MT873577
*Ascagilisguttulaspora*	MFLUCC 17-0244	NA	NG_064432	NA
*A.submersa*	MFLUCC 18-1143	NR_171970	MN888485	NA
*A.thailandensis*	MFLUCC 18-1149	NR_171969	MN913693	NA
*Botryosphaeriadothidea*	CBS 115476	NA	DQ678051	DQ767637
*Brachiosphaeratropicalis*	SS 2523	FJ887923	JN819284	JN819298
*Bullatosporiumtaxicola*	a21-004; CBS 151403	PP516536	PP516533	PP514386
*B.taxicola*	a21-005; CBS 151402	PP516535	PP516534	PP514385
*Capnodiumaciculiforme*	CBS 892.73	NA	GU301847	GU349045
*C.alfenasii*	CBS 146151	MN749233	MN749165	MN829346
*C.coartatum*	CPC 17779	MN749236	MN749167	MN829348
*Cenococcumgeophilum*	CG5	KC967409	NA	NA
*C.geophilum*	CG54	KC967410	NA	NA
*Chaetocapnodiuminsulare*	CBS 146159	NR_168830	NG_068681	MN829359
*C.philippinense*	MFLUCC 12-0110	NR_168831	KP744503	MN829362
*C.placitae*	CBS 124758	MH863403	MH874920	MN829363
*Conidiocarpusasiaticus*	MFLUCC10-0062	NA	JN832612	NA
*C.caucasicus*	GUMH937	NA	KC833050	NA
*C.fici-septicae*	MFLUCC 19-0072	MW063143	MW063206	NA
*C.siamensis*	SICAUCC 23-0010	OR405901	OR405912	OR671432
*Delitschiachaetomioides*	DSE871	MW209042	MW209067	MW238837
*D.winteri*	AFTOL-ID 1599	NA	DQ678077	DQ677922
	CBS 225.62			
*Dothideainsculpta*	CBS 189.58	NA	DQ247802	DQ471081
*D.sambuci*	DAOM 231303	NA	AY544681	DQ497606
*Ericboehmiacentramura*	chuni 70	KM272258	KM272256	KM277819
	MFLUCC 12-0808			
*E.curtisii*	CBS 198.34	NA	MH866967	FJ161093
*E.doimaeensis*	MFLUCC 16-0329	MH535872	MH535894	NA
*Fusculinaeucalypti*	CBS 120083	DQ923531	DQ923531	NA
*Gloniopsisarciformis*	GKM L166A	NA	GU323211	NA
*G.calami*	MFLUCC 15-0739	NR_164398	NG_059715	KX671965
*G.leucaenae*	MFLU 21-0201	OL782134	OL782050	OL875100
*G.percutanea*	FMR 8713	AM286786	LS997561	LS997569
*Gloniumcircumserpens*	CBS 123342	NA	FJ161208	NA
*G.circumserpens*	CBS 123343	NA	FJ161200	NA
*G.stellatum*	ANM 32; A. Miller 32, F	NA	GQ221887	GQ221926
*G.stellatum*	CBS 207.34	MZ570257	FJ161179	FJ161095
*Glyphiumelatum*	EB 0365; BPI 892671	KM220945	KM220939	KM220933
*Gordonomycesmucovaginatus*	CBS 127273	NR_157428	NG_057941	NA
*Graphylliumcaracolinense*	HUEFS 42838	NA	NG_060651	NA
*Guignardiagaultheriae*	CBS 447.70	MH859790	DQ678089	NA
*Halokirschsteiniotheliamaritima*	3124D	KM272366	NA	NA
*H.maritima*	CBS 221.60	NA	AY849943	GU349001
*H.maritima*	NWHC 45703-222	MK782369	NA	NA
*Hysteriumangustatum*	KUMCC 21-0213	OK482567	OK482568	NA
*H.pulicare*	EB 0238; CBS 123377	NA	FJ161201	FJ161109
*H.rhizophorae*	MFLUCC 15-0950	NR_189349	NG_241879	MF615401
*Hysterobreviumbaoshanense*	MFLUCC 16-2162	MZ467049	KX772765	KX772769
*H.constrictum*	KUN-HKAS102101	MN429070	MN429073	MN442088
*H.rosae*	CBS 149699	OQ990113	OQ990064	OQ989245
*Hysterodifractumpartisporum*	HUEFS 42865	NA	NG_060652	NA
*Hysterographiumdidymosporum*	MFLUCC 10-0101	NA	NG_064526	NA
*H.fraxini*	CBS 109.43	NA	FJ161171	FJ161088
*H.minus*	JCM 2758	NA	NG_059814	NA
*Hysteropatellaelliptica*	AFTOL-ID 1790	NA	DQ767657	DQ767640
	CBS 935.97			
*H.prostii*	G.M. 2016-02-20.2	MT341324	MT341324	NA
*Jahnulaappendiculata*	BCC11400	JN819280	FJ743446	JN819299
*J.dianchia*	KUMCC 17-0039	KY928456	KY928457	NA
*J.rostrata*	MFLU 20-0435	MT627720	MT627657	NA
*Lophiumarboricola*	CBS 758.71	NA	MH872091	NA
*L.arboricola*	FMR 3868	KU705825	KU705842	NA
*L.arboricola*	P99; KRAM F-59986	OR754902	OR754924	NA
*L.mytilinum*	CBS 114111	EF596819	EF596819	NA
*L.mytilinum*	CBS 269.34	OM337540	MH867013	NA
*L.zalerioides*	MFLUCC 14-0417	MF621583	MF621587	NA
*Massariainquinas*	WU 30527	HQ599402	HQ599402	HQ599342
*M.vomitoria*	WU 30606	HQ599437	HQ599437	HQ599375
*Mytilinidionacicola*	EB 0349; BPI 879794	NA	GU323209	NA
*M.acicola*	EB 0379; BPI 879793	NA	GU397346	NA
*M.andinense*	EB 0330; CBS 123562	NA	FJ161199	FJ161107
*M.australe*	CBS 301.34	NR_160067	MH867035	NA
*M.californicum*	EB 0385; BPI 879795	NA	GU323208	NA
*M.didymospora*	MFLUCC 16-0619	NA	MH535902	NA
*M.mytilinellum*	CBS 303.34	NA	MH867037	FJ161100
*M.mytilinellum*	EB 0386; BPI 879796 CBS 303.34	NA	GU397347	NA
*M.resinicola*	CBS 304.34	MH855535	MH867038	FJ161101
*M.rhenanum*	CBS 135.34	NA	NA	FJ161092
*M.rhenanum*	EB 0341; CBS 135.45	NA	GU323207	NA
*M.scolecosporum*	CBS 305.34	MH855536	MH867039	FJ161102
*M.thujarum*	EB 0268; BPI 879797	NA	GU323206	NA
*M.tortile*	CBS 306.34	MH855537	MH867040	NA
*M.tortile*	EB 0377; BPI 879798	NA	GU323205	NA
*Neocamarosporiumgoegapense*	CBS 138008	KJ869163	KJ869220	NA
*N.phragmitis*	MFLUCC 17-0756	MG844345	NG_070431	MG844351
*Neomassariafabacearum*	MFLUCC 16-1875	NA	KX524145	KX524149
*N.formosana*	NTUCC 17-007	NA	MH714756	MH714762
*Oedohysteriuminsidens*	ANM 1443	NA	GQ221882	NA
	A. Miller 1443, F			
*O.insidens*	CBS 238.34	NA	FJ161182	FJ161097
*O.sinense*	EB 0339; BPI 879800	NA	GU397348	GU397339
*Orbiliaauricolor*	AFTOL-ID 906	DQ491512	DQ470953	DQ471072
	CBS 547.63			
*O.vinosa*	AFTOL-ID 905	DQ491511	DQ470952	DQ471071
	CBS 917.72			
*Ostreichnionsassafras*	CBS 322.34	MH855548	FJ161188	NA
***Lijiangomyceslaojunensis***	**KUN-L 88703**	**PQ049177**	**PQ047633**	**PQ267963**
*Patellariaapiculatae*	MCD 096; MFLU 19-1236	MN047094	MN017860	NA
*P.atrata*	CBS 958.97	NA	GU301855	GU349038
*P.chromolaenae*	MFLUCC 17-1482	MT214381	MT214475	MT235796
*Pseudocamaropycnispini*	CBS 115589	KU728518	KU728557	
*Pseudocenococcumfloridanum*	Culture BA4b001	NA	LC095431	LC095383
	NBRC 111599			
	FLAS-F-59166			
*Psilogloniumaraucanum*	CBS 112412	NA	FJ161172	FJ161089
*P.colihuae*	MFLU 11-0214	KP744466	KP744511	NA
*P.macrosporum*	MFLU 18-2218	OR225075	OP612525	OR140436
*Purpurepitheciummurisporum*	MFLUCC 16-0611	NA	NG_059797	KY887666
*P.murisporum*	MFLUCC 17-0319	NA	KY799174	KY799177
*Quasiconchareticulata*	EB QR; RLG 14189	NA	GU397349	NA
*Quasiconcha* sp.	ZY 22.011	OR680490	OR680557	OR865892
	CGMCC 3.25498			
*Quasiconcha* sp.	ZY 22.012	OR680491	OR680558	OR865893
	CGMCC 3.25498			
*Quasiconcha* sp.	ZY 22.013	OR680492	OR680559	OR865894
	CGMCC 3.25498			
*Rhytidhysteronbannaense*	KUMCC 21-0483	OP526399	OP526409	OP572200
*R.bruguierae*	SDBR-CMU 473	OQ943970	OQ940376	OQ973477
*R.camporesii*	KUNCC 22-12388	OR807853	OR801302	OR832866
*Yuccamycescitri*	CBS 143161	MG386043	MG386096	NA
*Y.pilosus*	CBS 579.92	MG386044	MG386097	NA

The BLAST analysis of three genes (LSU, ITS, and mtSSU) from the specimen KUN-L 88687 indicated that this species belongs to Sclerococcales. Sequence data for all available taxa within this order were retrieved from GenBank and are listed in Table 2. Two species of *Caliciopsis* (Coryneliaceae, Coryneliales, Coryneliomycetidae, Eurotiomycetes) were included as the outgroup following Olariaga et al. (2019).

**Table 2.** Sequences used in phylogenetic analysis of Dactylosporaceae with specimens or strains’ information and GenBank accession numbers. Newly obtained sequences are in bold font. “NA” indicates the sequence is unavailable.

Species name	Voucher/strains	GenBank accession numbers		
		LSU	ITS	mtSSU
*Umbilicaria* sp.	INB_io4503Q	KM242300	KM242300	NA
*Umbilicaria* sp.	INB_io4513J	KM242356	KM242356	NA
*Umbilicaria* sp.	INB_io4513L	KM242358	KM242358	NA
*Caliciopsisorientalis*	CBS 138.64	NG_058741	NA	FJ190654
*C.pinea*	AFTOL-ID 1869	DQ678097	NA	FJ190653
	CBS 139.64			
*Cylindroconidiisaquaticus*	MFLUCC 11-0294	MH236579	MH236576	NA
*Fusichalaraminuta*	CBS 709.88	KX537758	KX537754	KX537762
*Gamsomycesaquaticum*	MFLUCC 18-1015	MN335230	MN335228	NA
*G.chiangmaiensis*	MFLUCC 18-0982	MN335229	MN335227	NA
*G.longisporus*	CBS 118.86	MT020877	MT020865	NA
*G.longisporus*	CBS 240.89	MT020878	MT020866	NA
*G.stilboideus*	CBS 146494	MT020879	MT020867	NA
*Pseudosclerococcumgolindoi*	ARAN-Fungi 6619	NG_073673	NR_171236	MK759897
*Rhopalophoraclavispora*	CBS 281.75	KX537756	KX537752	KX537760
*R.clavispora*	CBS 129.74	KX537755	KX537751	KX537759
*R.clavispora*	CBS 637.73	KX537757	KX537753	KX537761
*Sclerococcumahtii*	RP 23	KY661659	KY661630	KY661686
	F. Hognabba 1325a (H)			
*S.ahtii*	RP182	NA	KY661622	NA
	CHI17-37a (H)			
*S.chiangraiensis*	MFLU 16-0570	NG_066422	NR_163755	NA
*S.deminutum*	RP235	NA	KY661629	NA
	J. Pykala 39390 (H)			
*S.fusiformis*	MFLU 16-0593	NG_066423	NR_163756	NA
*S.fusiformis*	MFLU 18-0678	NA	MH718442	NA
*S.glaucomarioides*	RP275; Zhurbenko 13107 (LE 261065)	KY661660	KY661632	KY661683
*S.glaucomarioides*	KUN-L 88756	NA	OQ991232	OR035764
*S.haliotrephum*	AFTOL-ID 758	FJ176855	NA	KJ766382
	ATCC MYA-3590			
*S.haliotrephum*	J.K.5129B	FJ713617	NA	NA
	AFTOL-ID 798			
*S.lobariellum*	Diederich 18109	MH698499	NA	MH698503
*S.lobariellum*	Diederich 17708	MH698498	NA	MH698502
*S.lobariellum*	ARAN-Fungi 10091	MK759891	NA	MK759898
*S.mangrovei*	AFTOL-ID 2108	FJ176890	NA	KJ766383
*S.martynii*	D. Haelew. F_1567b	MZ221620	MZ221612	NA
	PUL F27737			
*S.martynii*	D. Haelew. F_1570b	MZ221623	MZ221616	NA
	PUL F27739			
*S.martynii*	D. Haelew. F_1577a	MZ221619	MZ221610	NA
	PUL F27741			
*S.parasiticum*	ARAN-Fungi 2724	MK759892	NA	MK759899
*S.parasiticum*	RP422	KY661666	KY661646	KY661690
	LE 260868			
*S.parasiticum*	F-283586	MK759894	NA	MK759901
*S.parasiticum*	F-283587	MK759895	NA	MK759902
*S.parasiticum*	ARAN-Fungi A3044025	MK759893	NA	MK759900
*S.pseudobactrodesmium*	CGMCC 3.25577T	OR514703	OR514694	OR588037
*S.pseudobactrodesmium*	GZCC 23-0056	OR514704	OR514695	OR588038
*S.pseudobactrodesmium*	GZCC 23-0057	OR514705	OR514696	OR588039
*S.pseudobactrodesmium*	GZCC 23-0549	OR514702	OR514693	OR588036
*S.ricasoliae*	A.F. 29132	MT153992	MT153963	MT153924
*S.ricasoliae*	A.F. 25967	MT153991	MT153962	MT153923
*S.ricasoliae*	A.F. Fla6b	MT153993	MT153964	MT153925
*S.ricasoliae*	A.F. 25611	MT153990	MT153961	MT153922
*S.simplex*	MFLU 21-0117	MZ655912	MZ664325	MZ676669
*Sclerococcum* sp.	A1153	MF071425	NA	MF085485
*Sclerococcum* sp.	A1016	KT263077	NA	KT263115
*Sclerococcum* sp.	RP391	KY661664	NA	KY661689
*S.sphaerale*	Diederich 17283	JX081673	NA	JX081678
*S.sphaerale*	Diederich 17279	JX081672	NA	JX081677
*S.sphaerale*	Ertz 17425 (BR)	JX081674	NA	JX081676
***S.stictae***	**KUN-L 88687**	**PQ407923**	**PQ408029**	**PQ415057**
***S.stictae***	**KUN-L 88687-1**	NA	**PQ408030**	NA
*S.tardum*	ICMP 24355	NA	NR_176187	NA
*S.tardum*	PDD 91756	NA	OL709435	NA
*S.tardum*	PDD 105454	NA	MK432753	NA
*S.stygium*	ARAN-Fungi 00823	NA	MK759886	MK759904
*S.stygium*	ARAN-Fungi 3395	MK759896	NA	MK759903
*S.stygium*	BHI-F312 (FH)	NA	MF161218	NA
*S.vrijmoediae*	NTOU 4002	KC692153	NR_138396	NA

**Abbreviations: ARAN-Fungi**: The ARAN-Fungi Fungarium; **BCC**: BIOTEC Culture Collection, Thailand; **BPI**: United States USDA ARS National Fungus Collections, Beltsville, MD; **CBS**: CBS Fungal Biodiversity Center, Utrecht, The Netherlands; **CGMCC**: China General Microbiological Culture Collection Center, Institute of Microbiology, Chinese Academy of Sciences, Beijing, China; **CPC**: Collection of P.W. Crous; **DAOM**: Canadian Collection of Fungal Cultures, Agriculture and Agri-Food Canada, Ottawa, Ontario, Canada; **FH**: FH Fungarium of Helsinki University Museum, Finland; **FLAS**: University of Florida Herbarium, located at the Florida Museum of Natural History in Gainesville, Florida, USA; **FMR**: Fungal Biodiversity Centre of the University of Valencia, Spain; **GZCC**: Guizhou Culture Collection, China; **GUMH**: Guangxi University Microbial Herbarium, China; **GZU**: Herbarium of the Institute of Botany, University of Graz, Austria; **HUEFS**: Herbário da Universidade Estadual de Feira de Santana, Brazil; **ICMP**: International Collection of Microorganisms from Plants, University of Auckland, New Zealand; **JAC**: University of Johannesburg Herbarium, USA; **JCM**: Japan Collection of Microorganisms; **KRAM**: Władysław Szafer Institute of Botany, Polish Academy of Sciences Herbarium; **KUMCC**: Kunming Institute of Botany Culture Collection; **KUN-HKAS**: Herbarium of Kunming Institute of Botany, Chinese Academy of Sciences, Yunnan, China; **KUN-L**: Lichen Herbarium of Kunming Institute of Botany, Chinese Academy of Science, Yunnan, China; **MFLU**: the herbarium of Mae Fah Luang University, Chiang Rai, Thailand; **MFLUCC**: Mae Fah Luang University Culture Collection, Chiang Rai, Thailand; **NBRC**: NITE Biological Resource Center, Japan; **NTOU**: National Taiwan Ocean University; **NWHC**: National Wildlife Health Center of U.S. Geological Survey; **PDD**: Plant Disease Database at the Auckland Museum, New Zealand; **PUFD**: Purdue University Forestry Department; **PUL**: Purdue University Herbarium; **RLG**: The Robert L. Gilbertson Mycological Herbarium at the University of Arizona; **SDBR-CMU**: Sustainable Development of Biodiversity Resources, Chiang Mai University, Thailand; **SICAUC**:Sichuan Agricultural University Culture Collection;**WU**: Herbarium of the University of Vienna.

Sequence alignment, concatenation, model selection, and format conversion were performed using the OFPT program (Zeng et al. 2023). Each gene region dataset was aligned using the ‘auto’ strategy in MAFFT (Katoh and Standley 2013) and trimmed with the ‘gappyout’ command in TrimAl (Capella-Gutierrez et al. 2009). The best-fit nucleotide substitution models for each dataset were selected using the Bayesian Information Criterion (BIC) from twenty-two common DNA substitution models with rate heterogeneity, as implemented in ModelFinder (Kalyaanamoorthy et al. 2017). The datasets were then concatenated with partition data for subsequent phylogenetic analyses.

Maximum likelihood (ML) analysis was conducted on the IQ-TREE web server applying the ultrafast bootstrap approximation with 1,000 replicates (Hoang et al. 2018), and the SH-like approximate likelihood ratio test (SH-aLRT) (Guindon et al. 2010; Nguyen et al. 2015). The consensus tree was summarized using the extended majority rule. For further verification, an additional ML analysis was performed with RAxML-HPC2 on ACCESS (v8.2.12), using the GTRGAMMA model with a rapid bootstrap analysis of 1000 replicates (Miller et al. 2010; Stamatakis 2014).

Bayesian inference was carried out using two parallel Metropolis-coupled Markov Chain Monte Carlo (MCMC) runs, each consisting of one ‘cold’ chain and three heated chains, in MrBayes (Ronquist et al. 2012). Trees were sampled every 1,000 generations, and the run was terminated when the average standard deviation of split frequencies fell below 0.01. The final tree was summarized after discarding the first 25% of samples as burn-in and visualized in FigTree v1.4.4 (Rambaut 2016). The newly identified taxon was registered in Index Fungorum and Faces of Fungi database (Jayasiri et al. 2015).

## ﻿Results

**Figure 1.**
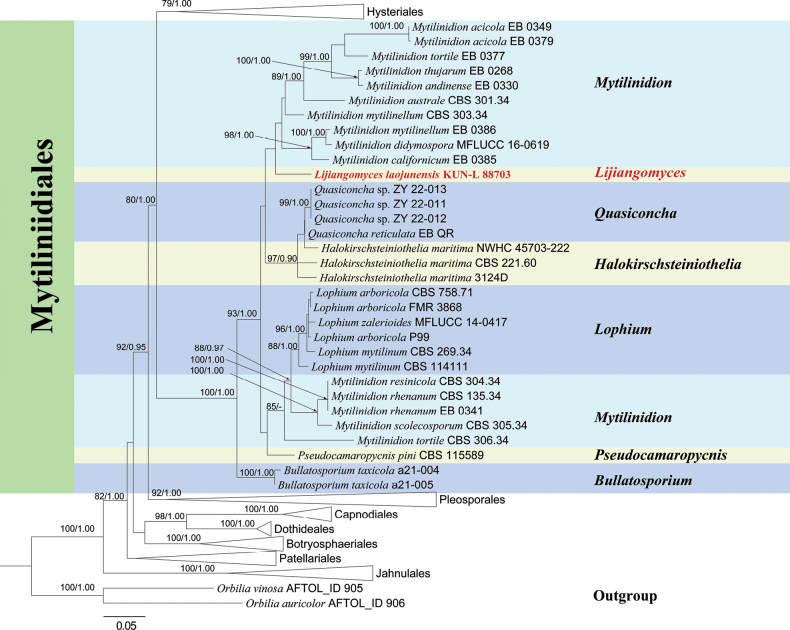
RAxML analysis of Mytilinidiales based on the combined LSU, ITS, and *tef*1-α sequence data. Bootstrap support values for maximum likelihood (ML ≥ 70%), and the Bayesian Posterior Probabilities (PP ≥ 0.90) are shown near the nodes as ML/PP. *Orbiliaauricolor* (AFTOL-ID 906) and *O.vinosa* (AFTOL-ID 905) were used as outgroups. The newly generated sequence is in red bold font.

In the analysis of Mytilinidiales, the final dataset comprised 116 taxa (Table 1) with 2246 aligned characters, including gaps (ITS 1–494 bp, LSU 495–1346 bp, and *tef*1-α 1347–2246 bp). The best-fit models for each gene, determined using the Bayesian information criterion (BIC), were as follows: ITS: SYM+I+G4, LSU: TN+F+R4, and *tef*1-α: TN+F+I+G4. For the combined dataset (LSU, ITS, and *tef*1-α), the parameters of the GTRGAMMA model were as follows: estimated base frequencies: A = 0.24, C = 0.25, G = 0.28, T = 0.23; substitution rate: AC = 1.12, AG = 3.35, AT = 1.77, CG = 0.88, CT = 8.54, GT = 1.00; gamma distribution shape parameter (α) = 0.325118; and tree-length = 6.868758. The best-scoring RAxML tree was constructed with a final maximum likelihood (ML) optimization likelihood value of -34,803.39. Bayesian posterior probabilities (BYPP) were calculated using MCMC analysis, achieving a final average standard deviation of split frequencies of 0.009998. The final tree topologies of ML and BYPP analyses were consistent. The best-scoring RAxML tree, based on combined LSU, ITS, and *tef*1-α sequence datasets, is presented in Fig. 1.

The resulting phylogram distinguishes eight order-level clades, most of which are well-supported, except for the Patellariales clade. Seven genera form a strongly supported Mytilinidiales clade, with *Mytilinidion* appearing polyphyletic. The newly identified species clusters within the Mytilinidiales clade are closely related to a *Mytilinidion* subclade containing eight species. The remaining *Mytilinidion* species form a separate clade alongside *Lophium*. *Halokirschsteiniothelia* and *Quasiconcha* form a sister clade to the *Ostreola-Mytilinidion* grouping. Additionally, two *Bullatosporium* strains form a distinct clade closely related to other genera within the Mytilinidiales order.

**Figure 2.**
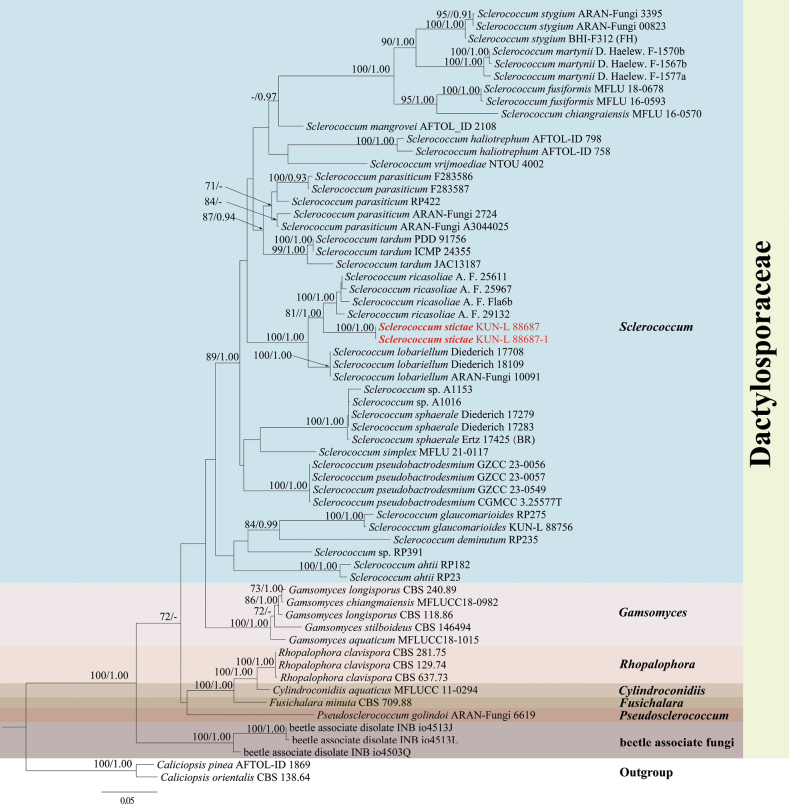
RAxML analysis of Dactylosporaceae based on the combined LSU, ITS and mtSSU sequence data. Bootstrap support values for maximum likelihood (ML ≥ 70%), and the Bayesian Posterior Probabilities (PP ≥ 0.90) are shown near the nodes as ML/PP. *Caliciopsisorientalis* (CBS 138.64) and *Caliciopsispinea* (AFTOL-ID 1869) were used as outgroups. The newly generated sequence is in red bold font.

In the analysis of Dactylosporaceae, the final dataset comprised 62 taxa (Table 2) with 2041 aligned characters, including gaps (ITS 1–455 bp, LSU 456–1310 bp, and mtSSU 1311–2041 bp). The best-fit nucleotide substitution models, selected based on the Bayesian information criterion (BIC), were as follows: ITS: TIM2e+I+G4, LSU: TNe+I+G4, mtSSU: TVM+F+I+G4. The parameters for the GTRGAMMA model of the combined LSU, ITS, and mtSSU were as follows: estimated base frequencies: A = 0.29, C = 0.19, G = 0.25, T = 0.26, and substitution rate AC = 1.01, AG = 2.68, AT = 1.67, CG = 0.85, CT = 5.43, and GT = 1.00. Gamma distribution shape parameter α = 0.256278, and the tree length = 2.405501. The best-scoring RAxML tree was constructed with a final ML optimization likelihood value of - 14773.67. The final tree topologies of ML and BYPP analyses were consistent. The best-scoring RAxML tree, based on combined LSU, ITS, and mtSSU sequence datasets, is presented in Fig. 2. The newly collected specimen clusters within the *Sclerococcum* clade and is closely related to *S.ricasoliae*.

### ﻿Taxonomy

#### ﻿ Lijiangomyces

Taxon classificationFungi

Meng & Jayaward. gen. nov.

B790F3D6-BBD2-57D7-8FC6-01A068A7ACC6

Index Fungorum: IF903174

Facesoffungi Number: FoF17069

##### Etymology.

The genus name “*Lijiangomyces*” refers to “Lijiang”, the city in Yunnan Province of China from where the holotype was collected.

##### Description.

***Sexual morph*: *Ascomata*** hysterothecia, superficial, solitary, dispersed, sessile, obovoid to broadly shell-shaped or irregularly rounded, with a broadly open or slit-like disc. ***Margin*** black, vertically erect, fragile, with the disc surface appearing yellowish-brown. ***Peridium*** carbonaceous, black in the lateral and upper regions. ***Hymenium*** hyaline to slightly yellowish, with a densely packed hamathecium. ***Paraphyses*** filiform, hyaline, non-anastomosed, and non-septate. ***Hypothecium*** slightly yellowish. ***Asci*** bitunicate, 8-spored, elongated to clavate, with a rounded apex lacking ascal wall thickening, I-, K-. ***Ascospores*** uniseriate, arranged obliquely and parallelly, hyaline, thin-walled, smooth, fusiform to ellipsoidal, K-, I+ dark blue, aseptate at immature, becoming muriform at maturity with 4–7 transverse septa and 1–2 longitudinal septa. ***Asexual morph***: Not observed.

##### Type species.

*Lijiangomyceslaojunensis* Meng & Jayaward.

##### Notes.

The genus is distinguished by black, broadly shell-shaped to irregularly rounded hysterothecial ascomata, with a broadly open or occasionally closed disc, typically light brown to flesh-yellow. Asci are elongated to clavate, containing eight uniseriate, hyaline, and muriform ascospores. Phylogenetic analysis places this genus within the family Mytilinidiaceae (Mytilinidiales, Dothideomycetes) closely related to *Mytilinidion*. In the single-gene phylogenies, the new species is positioned outside the *Mytilinidia* sensu stricto clade in the ITS and LSU trees but clusters within it in the *tef*1-α tree. However, in the concatenated analysis combining all three genes, it is again placed outside the *Mytilinidia* sensu stricto clade. These results support its recognition as a distinct lineage within Mytilinidiaceae. Morphologically, this genus differs significantly from *Mytilinidion* in having obovoid to broadly shell-shaped or irregularly rounded, with broadly open ascomata (vs. globoid to obovoid, conchate, or dolabrate ascomata with narrow slit-like openings) and hyaline and muriform ascospores (vs. hyaline to brown and transverse septa). In addition, this genus shares similar morphology with *Ostreola* in having muriform ascospores but differs in the broadly shell-shaped or irregularly rounded ascomata (vs. conchiform to hatchet-shaped), a broad disc opening (vs. narrowly slit-like), and hyaline ascospores (vs. brown).

#### ﻿ Lijiangomyces laojunensis

Taxon classificationFungi

Meng & Jayaward. sp. nov.

FA6618B1-0759-52D9-9A44-16136E928852

Index Fungorum: IF902457

Facesoffungi Number: FoF16265

Fig. 3

##### Etymology.

The species epithet “*laojunensis*” refers to the type locality “Laojun Mountain National Nature Reserve” in Yunnan Province of China.

**Figure 3.**
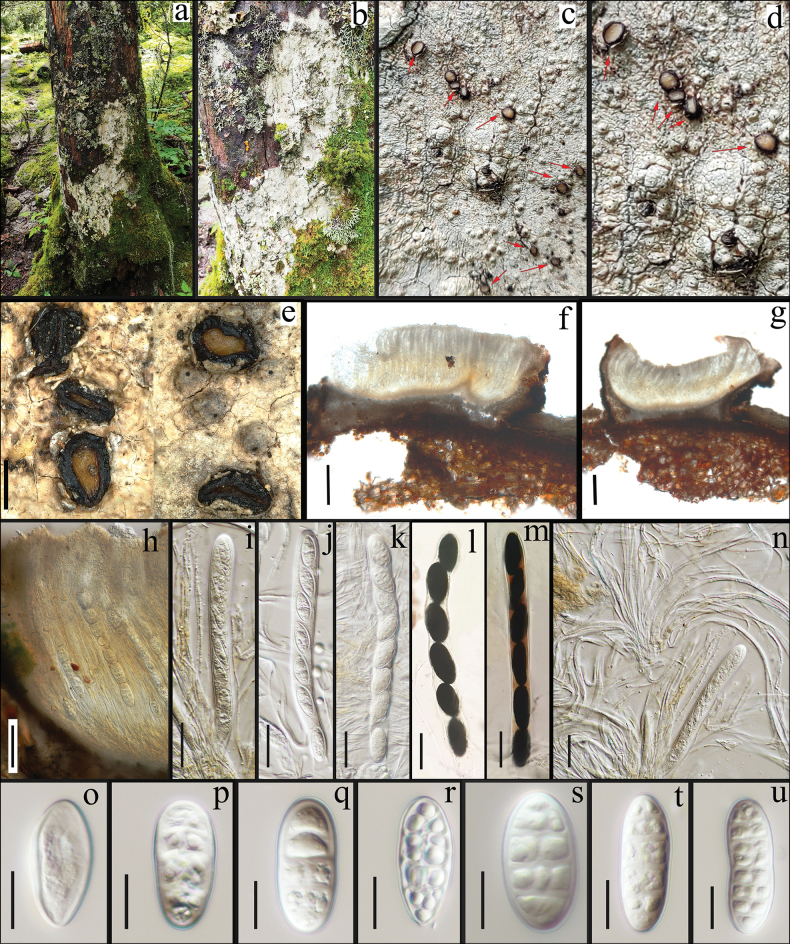
*Lijiangomyceslaojunensis* sp. nov., (KUN-L 88703) **a, b** habitat **c–e** ascomata (arrows) **f–h** section of ascomata in water **i–k** asci in water **l, m** asci in IKI **n** paraphyses in water **o–u** ascospores in water. Scale bars: 500 μm (**e**); 200 μm (**f, g**); 50 μm (**h**); 25 μm (**i–n**); 10 μm (**o–u**).

##### Holotype.

KUN-L 88703.

##### Description.

***Sexual morph*: *Ascomata*** hysterothecia, (0.8–)0.88–1.05(–1.1) × (0.4–)0.47–0.78(–0.8) mm (x– = 0.97 × 0.63, n = 10), superficial, solitary, dispersed, sessile, non-stromatic, obovoid to broadly shell-shaped or irregularly rounded, with a broadly open or slit-like disc. ***Margin*** black, vertically erect, fragile, with a yellowish-brown, slightly depressed disc surface appearing below the rim of the lateral wall. ***Peridium*** 70–100 μm thick, carbonaceous, black laterally and apically, transitioning to grayish near the base. ***Hymenium*** 350–400 μm high, hyaline to slightly yellowish, densely packed with hamathecium. ***Paraphyses*** 1–2 μm wide, unbranched, hyaline, non-anastomosed, non-septate. ***Hypothecium*** 35–50 μm thick, slightly yellowish. ***Asci*** (120–)122.6–168.7(–190) × (13–)13.4–16.5(–18) μm (x– = 145.7 × 14.9, n = 10), bitunicate, 8-spored, elongated to clavate, rounded apex, without apical thickening of ascal wall, K-, I-. ***Ascospores*** (20–)20.9–28.8(–37.5) × (9–)10.1–15.4(–17.5) (x– = 24.8 × 12.8, n = 30) μm, K-, I+ reddish brown, then turning to dark blue, uniseriate, arranged obliquely and parallelly, hyaline, thin-walled, smooth, fusiform to ellipsoidal, aseptate at immature, becoming muriform at maturity with 4–7 transverse septa and 1–2 longitudinal septa, sometimes slightly constricted at the median septum, rounded at the ends in aged ascospores. ***Asexual morph***: Not observed.

##### Material examined.

China • Yunnan Province, Lijiang City, Laojun Mountain National Nature Reserve, 26°39'N, 99°43'E, 3900 m elev., on the bark of *Abiesfabri* (Pinaceae), 10 Apr 2022, Qing-feng Meng, ljs-52 (holotype KUN-L 88703).

##### Notes.

*Lijiangomyceslaojunensis* closely resembles *Ostreolaconsociata* (the type species of *Ostreola*) and *O.sessilis*, in having cylindrical asci and uniseriate muriform ascospores. However, the new species is distinguished by its ascomatal morphology, which is broadly shell-shaped or irregularly rounded with a widely opened disc, in contrast to *Ostreola* species which have conchiform to hatchet-shaped ascomata with a narrow slit-like opening. Furthermore, the ascospores of the new species are hyaline and larger in size (20.9–28.8 × 10.1–15.4 μm), in contrast, brown and smaller ascospores (14–22 × 6–8 μm) are the characteristic feature of *Ostreola* (Darker 1963).

Phylogenetic analysis places this species as a sister clade to *Mytilinidion*, however, it can be distinguished by its obovoid to broadly shell-shaped or irregularly rounded ascomata (vs. globoid to obovoid, erect, conchate, or dolabrate), and hyaline and muriform ascospores (vs. hyaline to dark brown and transversely septate) (Boehm et al. 2009b; Jayasiri et al. 2018).

#### ﻿ Sclerococcum stictae

Taxon classificationFungi

Meng, Diederich & Thiyagaraja sp. nov.

70A64039-B13C-5A8F-96F4-1D18FA6292DC

Index Fungorum: IF903175

Facesoffungi Number: FoF17070

Fig. 4

##### Etymology.

The species epithet “*stictae*” refers to “*Sticta*”, the host lichen on which the holotype was found.

**Figure 4.**
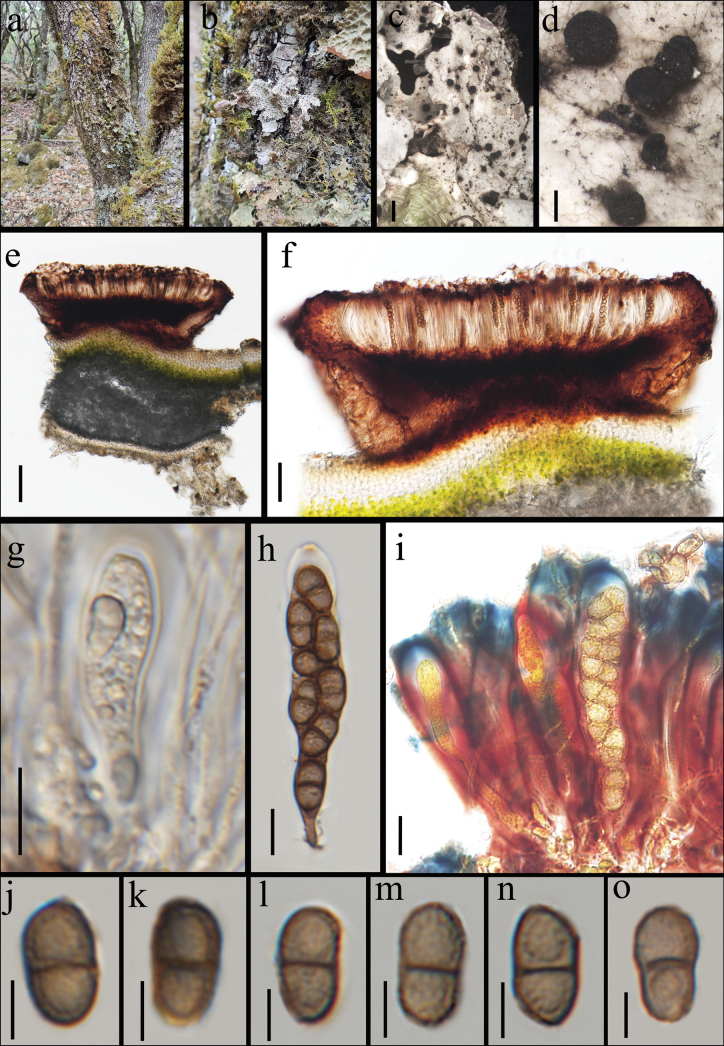
*Sclerococcumstictae* sp. nov., growing on the thallus of *Sticta* sp. (KUN-L 88687) **a, b** habitat and appearance of the host lichen **c, d** appearance of the apothecia **e, f** section of apothecium in water **g** immature ascus in water **h** mature ascus with ascospores in water **i** asci in I **j–o** ascospores in water. Scale bars: 1 mm (**c**); 200 μm (**d**); 100 μm (**e**); 50 μm (**f**); 10 μm (**g–i**); 5 μm (**j–o**).

##### Holotype.

KUN-L 88687.

##### Description.

***Sexual morph*: *Ascomata*** apothecioid, 200–300 μm in diam., rounded, cup-shaped, sessile, erumpent from the host thallus with a narrow base, 170–200 μm in diam., either dispersed or occurring in small groups, black, matte. ***Disc*** flat, black. ***Margin*** distinct, persistent, and concolorous with the disc. ***Exciple*** brown, paraplectenchymatous, laterally 40–70 μm wide. ***Epithecium*** (12–)14.3–21.0(–24) μm thick (x– = 17.7, n = 30), with dark brownish granules. ***Hymenium*** (46–)65.2–94.8(–96) μm high (x– = 80, n = 30), light brown, and distinctly gelatinized, K/I+ reddish with bluish in epihymenium. ***Paraphyses*** 2–3 μm wide, anastomosing, branched, septate, with swollen, pigmented apices. ***Hypothecium*** (86–)89.6–95.2(–97) μm thick (x– = 92.4, n = 30), dark orange-brown, with irregularly shaped hypothecial cells. ***Asci*** (40–)44.8–67.9(–72) × (7–)7.8–11.9(–12) μm (x– = 56.3 × 9.9, n = 10), bitunicate, narrowly clavate to cylindrical, ascus wall thickened at the apex, ocular chamber absent, 8-spored, K/I-, except for the K/I+ blue outer gelatinous coat, most intensely colored around the ascus apex. ***Ascospores*** (8–)9.2–11.1(–12) × (5–)5.1–6.2(–7) μm (x– = 10.1 × 5.6, n = 30), l/w ratio = (1.5–)1.7–1.9(–2) (x– = 1.8, n = 30), brown when mature, 1-septate, slightly constricted at the septum, verrucose, slightly asymmetric with a larger upper cell, ellipsoidal, sometimes soleiform. ***Asexual morph***: Not observed.

##### Material examined.

China • Yunnan Province, Diqing Autonomous Prefecture, Meili Mountain National Nature Reserve, 28°24'N, 98°48'E, 3300 m elev., on the thallus of *Sticta* sp., on the bark of *Rhododendronlapponicum* (Ericaceae), 21 Apr 2023, Qing-feng Meng, ml-68 (holotype KUN-L 88687).

##### Notes.

*Sclerococcumstictae* clusters within a well-supported subclade along with *S.ricasoliae* and *S.lobariellum*. The basepair comparison with *S.ricasoliae* revealed 7.5% differences (34/455 bp) in ITS, 2.2% (19/855 bp) differences in LSU, and 1% (7/730 bp) differences in mtSSU sequences (Flakus et al. 2019). Compared to *S.lobariellum*, it exhibits 4.21% differences (36/855 bp) in LSU and 1.1% (8/730) in mtSSU. Morphologically, *S.stictae* resembles *S.ricasoliae* in its ascomatal and ascospore appearance but can be distinguished by its longer, narrower asci (c. 45–68 × 8–12 μm vs. 35–50 × 10–15 μm) and broader ascospores (c. 9–11 μm vs 4–6 μm), with a smaller length/width ratio (1.7–1.9 vs. 1.5–3.5) (Flakus et al. 2019). Phylogenetically, *S.stictae* is also related to *S.lobariellum*, but the basepair comparison revealed 4.3% (37/855 bp) differences in LSU and 1.1% (8/730 bp) differences in mtSSU sequences (Hafellner 1979). Hence based on recommendations outlined by Jeewon and Hyde (2016), the establishment of the new species is supported. Diederich et al. (2024) have shown that both *Sclerococcumlobariellum* and *S.ricasoliae* possess an asexual stage producing dark brown, dispersed, muriform conidia, often co-occurring with the sexual stage. In all other known lichenicolous *Sclerococcum* species with an asexual stage, conidia are produced within compact sporodochia, and this stage is never accompanied by a sexual stage. We anticipate, therefore, that an asexual stage with dispersed, muriform conidia also exists in the new *S.stictae* and should be searched for when more specimens become available.

Another species, *Sclerococcumdendriscostictae*, also found on *Sticta*, shares morphological traits with *S.stictae*. However, the new species can be distinguished by its longer asci (c. 45–68 × 8–12 μm vs. 33–44 × 9.5–13.5 μm) and verrucose ascospore ornamentation, in contrast to smooth-walled ascospores reported in *S.dendriscostictae* (Joshi 2021).

## ﻿Discussion

Hysteriaceous fungi are distinguished by their persistent, carbonaceous, navicular pseudothecia with a longitudinal slit opening. Historically, the mytilinidiaceous fungi, which possess fragile, shell-shaped pseudothecia that dehisce through a longitudinal cristate apex, were considered part of the hysteriaceous. However, Boehm et al. (2009a, 2009b) reclassified these fungi based on phylogenetic analyses, leading to the establishment of the order Mytilinidiales.

In our phylogenetic analysis, six genera within Mytilinidiales form a distinct yet complex clade. *Mytilinidion* is split into two subclades, indicating its polyphyletic nature. This is consistent with the previous study by Boehm et al. (2009a). One subclade includes four species of *Mytilinidion* alongside all *Lophium* species, forming a well-supported *Lophium-Mytilinidion* clade. The other subclade contains eight *Mytilinidion* species with our new collection, although this grouping is weakly supported. Morphologically, *Mytilinidion* species are defined by globoid to obovoid, conchate, or dolabrate ascomata with narrow slit-like openings, bitunicate, 8-spored asci, and hyaline to dark brown ascospores with 3–5(–7) transverse septa (Boehm et al. 2009b; Jayasiri et al. 2018). However, our new species displays distinct morphological features that diverge significantly from those of *Mytilinidion*, supporting its exclusion from this genus based on both phylogenetic and morphological evidence.

Hyde et al. (2024) accepted one family and eleven genera within Mytilinidiales, most of which lack muriform septation except *Ostreola* which was introduced by Darker (1963). Although molecular evidence for *Ostreola* remains unavailable, our new species shares features with it, such as cylindrical asci and uniseriate muriform ascospores but differs from *Ostreola* in having broadly opened hysterothecia, while *Ostreola* exhibits narrow, slit-like openings. Additionally, *Ostreola* typically has dull-brown muriform spores, whereas our species is distinguished by its hyaline muriform ascospore (Harkness and Cooke 1878; Darker 1963; Tilak and Kale 1968; Rao and Modak 1972; Barr 1987).

Phylogenetic analysis further positions our new species at a significant distance from the recently described genus *Bullatosporium* (Andreasen et al. 2024). Apart from the six genera included in our phylogenetic analysis, four additional genera within the family, *viz. Actidium*, *Camaroglobulus*, *Peyronelia*, and *Zoggium* lack molecular data but exhibit distinct morphological characteristics. *Actidium* is characterized by simple, rounded spores (Fries 1815). *Camaroglobulus* was introduced as the asexual morph of *Mytilinidionresinae*, though its taxonomic placement requires further molecular confirmation (Speer 1986). *Peyronelia* is defined by brown, fusiform conidia that form short chains of slender, septate, interconnected cells (Ciferri and Fragoso-Romualdo 1927) and *Zoggium* features broadly filiform or vermiform spores that are transversely septate and pale-colored (Vasilyeva 2001).

Given these morphological and phylogenetic distinctions, the establishment of a new genus to accommodate our newly identified species is both necessary and justified. This classification will provide a clearer framework for understanding diversity within the order Mytilinidiales.

The holotype of *Lijiangomyceslaojunensis* exhibits another intriguing feature: the base of its apothecium is surrounded by the thallus of *Pertusaria* sp. This phenomenon has led us to mistakenly identify it as a lichenicolous species. Although lichenicolous behavior was not confirmed in this study, the close physical association suggests that the two fungi coexist without conflict. It is also possible that lichenicolous species may be identified as more specimens are collected and studied in the future.

Before 2018, *Dactylospora* and *Sclerococcum* were considered distinct genera, with *Sclerococcum* containing 21 species, 19 of which were lichenicolous. Diederich et al. (2018) synonymized *Dactylospora* with *Sclerococcum* and transferred 46 species from *Dactylospora* to *Sclerococcum*. Subsequently, Olariaga et al. (2019) transferred 14 non-lichenicolous species of *Dactylospora* to *Sclerococcum*. Johnston (2022) introduced six new saprophytic species of *Sclerococcum*. Since 2018, ten new lichenicolous species and three combinations have been added to the genus (Elix et al. 2019; Flakus et al. 2019; Fryday 2019; Navarro-Rosinés and Romero 2019; Spribille et al. 2020; Joshi 2021; Zhurbenko 2022; Paz-Bermúdez et al. 2023; Diederich et al. 2024; Zhurbenko and Diederich 2024). Diederich et al. (2024) have accepted a total of 85 species in *Sclerococcum*, with 64 lichenicolous species and the remainder being saprotrophs on liverworts, wood, and bark in both terrestrial and marine habitats (Dong et al. 2020; Thiyagaraja et al. 2022).

The first study of *Sclerococcum* in China was conducted by Thiyagaraja et al. (2022), who reported a new geographical record of *S.simplex*, collected from a corticolous *Pertusaria* thallus in Yunnan province. Ma et al. (2023) described the lignicolous asexual species, *S.pseudobactrodesmium* from Guizhou Province. Meng et al. (2024) reported a new geographical record of *S.glaucomarioides* found on *Ochrolechiaakagiensis* from China. This study provides an additional new *Sclerococcum* species from China.

## Supplementary Material

XML Treatment for Lijiangomyces

XML Treatment for Lijiangomyces laojunensis

XML Treatment for Sclerococcum stictae

## ﻿Additional information

### Author ORCIDs

Qingfeng Meng https://orcid.org/0000-0001-9814-8238

Paul Diederich https://orcid.org/0000-0003-0357-7414

Vinodhini Thiyagaraja https://orcid.org/0000-0002-8091-4579

Damien Ertz https://orcid.org/0000-0001-8746-3187

Xinyu Wang https://orcid.org/0000-0003-2166-6111

Natsaran Saichana https://orcid.org/0000-0002-4357-7980

Kevin D. Hyde https://orcid.org/0000-0002-2191-0762

Ruvishika S. Jayawardena https://orcid.org/0000-0001-7702-4885

Shaobin Fu https://orcid.org/0000-0001-9932-1346

### Data availability

All of the data that support the findings of this study are available in the main text.
